# Recent advancements in the control of *Taenia solium*: A systematic review

**DOI:** 10.1016/j.fawpar.2018.e00030

**Published:** 2018-11-13

**Authors:** T. de Coster, I. Van Damme, J. Baauw, S. Gabriël

**Affiliations:** Ghent University, Faculty of Veterinary Medicine, Department of Veterinary Public Health and Food Safety, Salisburylaan 133, 9820 Merelbeke, Belgium

**Keywords:** Taenia solium, *Cysticercosis*, *Taeniosis*, *Systematic review*, *Control*, *Elimination*

## Abstract

The combined health and economic impact of *Taenia solium* urges for control and, if possible, elimination of this neglected parasitic zoonosis. Up till now there is still no consensus about the most cost-effective and feasible approaches for control. The objective of this systematic review is to identify and summarize the evidence in English scientific literature on the control and elimination of *T*. *solium* since 2014, based on the rapidly evolving field of evidence on control and elimination of *T*. *solium*. The search resulted in the identification of 458 records of which 31 were included, covering 13 field trials and 18 articles containing experimental data, mathematical models, and other information directly relevant the control of *T*. *solium*.

Recent field studies confirm that combinations of interventions or multiple rounds are more successful in obtaining rapid reductions in transmission and parasite occurrence, with the quick impact of the combination of human and pig treatment confirmed in a South Asian and Peruvian context. Moreover, elimination of transmission through a one-year intensive program, combining human and pig treatment/vaccination was described in a Peruvian study. Recent studies also provide more data on the positive impact of specific health education, as well as newly developed electronic educational tools, providing opportunities for area specific community-engaged participatory interventions.

Once control has been achieved, monitoring of migration of both potentially infected people and pigs from outside the control area is important for sustained disease control.

## Highlights

• Combined intervention field studies demonstrate sharp decreases in disease occurrence.
• New, specific, efficient, electronic educational tools are available.
• Elimination of *Taenia solium* in a Peruvian endemic area has been described.
• Integration of *Taenia solium* control in other NTD control programs can be effective.

## 1 Introduction

*Taenia solium* is a leading cause of acquired epilepsy in endemic areas (Ndimubanzi et al., 2010). In 2014, the parasite was ranked first on the global scale of foodborne parasites (WHO/FAO, 2014). This parasite zoonoses has human as final host, carrying the tapeworm (taeniosis, TS), pigs as normal intermediate hosts with the metacestode larval stages (cysticerci, porcine cysticercosis, PCC), while human can also act as accidental intermediate host (human cysticercosis, HCC). In the latter case, the cysticerci have a tendency to settle in the central nervous system, causing neurocysticercosis (NCC), leading to neurological signs and symptoms (Murrell, 2005). Besides epileptic seizures, chronic headaches are a common symptom of NCC (Carabin et al., 2011). The neglected parasitic zoonosis is most common in regions associated with poverty, lack of sanitation and free-range pig husbandry, allowing direct contact with human faecal material, combined with an insufficient implementation of meat inspection and safe consumption of meat (Murrell, 2005; Kyvsgaard et al., 2007; Coral-Almeida et al., 2015).

*Taenia solium* was recently re-estimated as a leading cause of deaths from foodborne diseases, with a total of 2.8 million disability-adjusted life-years (DALYs) a year in low and middle income countries related to human cysticercosis (WHO, 2015a). Considerable economic losses in the public health and agricultural sector exists as well. These are mainly due to NCC, causing the loss of wage-earning activities (Urajkotia et al., 2007). Annual losses due to PCC have been estimated at 25 million USD for ten West and Central African countries (Zoli et al., 2003) while on average 5 million USD were attributed to agricultural losses in Eastern Cape Province, South Africa (Carabin et al., 2006).

The combined health and economic impact urges for control and, if possible, elimination of this zoonosis. The first practical recommendations upon control of *T*. *solium* date back to 1976 (FAO/UNEP/WHO, 1977), followed by the ‘potential eradicable’ declaration in 1993 by the International Task Force on Disease Eradication (ITFDE) (Schantz et al., 1993). Adjustments to this statement were made twice (ITFDE, 2003; Center, 2013), with the challenges identified in 2013 such as the lack of routine surveillance and reporting, the need for rapid diagnostic tests and the need for data of how preventative chemotherapy affects prevalence (Center, 2013) still remaining (Thomas, 2015; Gabriël et al., 2016).

The World Health Organisation (WHO) highlighted *T*. *solium* cysticercosis as a Neglected Zoonotic Disease (NZD) (WHO, World Health Organization, 2006, WHO, World Health Organization, 2007, WHO, World health organization, 2010) and included the parasite in 2012 in the road map to tackle the Neglected Tropical Diseases (NTDs). This road map targeted a validated, stepwise approach for the control and elimination of *T*. *solium*, using the most cost-effective control tools to be ready and available by 2015, with elimination interventions scaled up in selected countries from 2016 to 2020 (WHO, World Health Organization, 2012, WHO, World Health Organization, 2015b). The international community also pledged their commitment to this goal in the London Declaration (WHO, 2013) and World Health Assembly Resolution WHA66.12 also requested member states, international partners and the Director General WHO to provide support for the activities outlined in this road map. However, the 2015 goal has not been met (WHO, 2017).

**Fig. 1:**
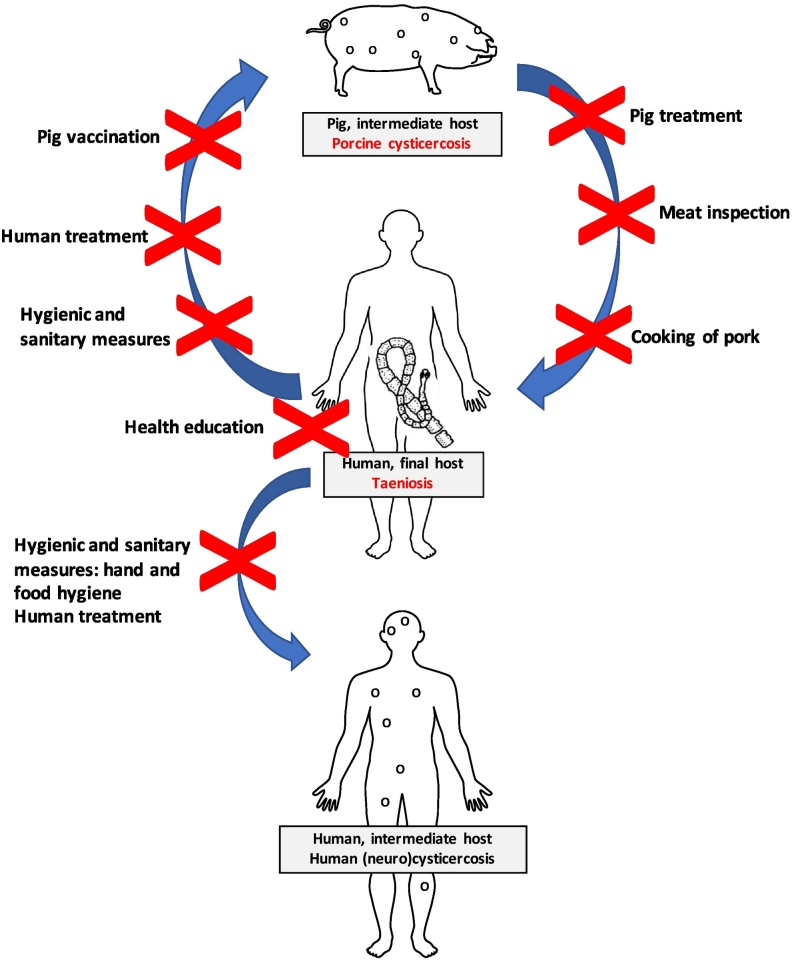
Life cycle of *Taenia solium* and possible interventions (X) which are considered to interrupt the life cycle in endemic areas.

Available control measures (Fig. 1) can be divided into measures on human level, targeting the final host and measures on pig level, targeting the intermediate host. The first group, targeting the human final host, include health education, hygienic and sanitary improvements and human treatment. A second group of control measures, targeting the pig intermediate host includes meat inspection and proper meat cooking, pig husbandry and pig treatment and/or vaccination. A landscape analyses describing results from studies investigating all these intervention types was conducted by Thomas (2015). In recent years, a substantial amount of new information has become available. The objective of this systematic review is to identify and summarize the recent evidence on the control and elimination of *T*. *solium* since the systematic review by Thomas (2015).

As a result, this review will render a complete and actual overview of the recent accessible information and evidence on the rapidly evolving field of control and elimination of *T*. *solium*.

## 2 Materials and methods

The systematic review was conducted following the principles of the PRISMA guidelines.

### 2.1 Review question

This review aims to identify, collect and assess all relevant articles published after 01 January 2014 in English scientific literature, including empirical data as well as evidence relating to aspects of neglected tropical disease (NTD) control which are directly relevant to the control and elimination of *T*. *solium*. The review question was the following: ‘Is there new evidence on the efficacy and development of existing or new tools for the control or elimination of *T*. *solium* since 2014?’

The identifying key elements of the question using the PICOT acronym where as follows:

- **P**opulation: humans or pigs.

- **I**ntervention: drugs (Praziquantel, niclosamide, albendazole, mass drug administration, TSOL18, vaccination, oxfendazole), education, latrines, sanitation, husbandry or other.

- **C**omparator: non-treated, local/experimental study population.

- **O**utcome: efficacy, side effects, acceptance, costs, risk factors.

- **T**imeframe: From 1st January 2014 till 1st May 2017, supplementary articles till 1st March 2018.

### 2.2 Search methods and exclusion criteria

The search was performed in twofold by two independent researchers (TDC, JB). The following search engines were used: IngentaConnect, PubMed, Library of Congress, British Library, ScienceDirect, African Journals Online and Google Scholar, applying a combination of the following search terms: 1st January 2014 (2014/01/01, date of publication) AND *Taenia solium* OR cysticercosis OR taeniasis AND control OR elimination. All searches were performed within titles and abstracts. Afterwards, duplicates were removed and the articles were screened first on title, secondly on abstract and finally on full text. Exclusion of non-fitting papers were made according to the following criteria: Studies published before 1st January 2014, studies not relating to humans or pigs, studies not relating to NTDs, studies on aspects of NTDs which do not discuss issues relevant to *T*. *solium* control/elimination (e.g. no control intervention), papers relating to clinical symptoms, diagnoses and treatment of NCC including case studies, purely epidemiological studies on *T*. *solium*, papers on diagnoses of *T*. *solium* cysticercosis/TS (including diagnostic imaging), papers on aspects of basic sciences (immunology/molecular biology/physiology/bio chemistry/basic pharmacology), studies about control/elimination in Europe/US, papers not written in the English language and reviews. At last additional resources were identified by accessing citations within selected papers and by implementation of articles published after the initial search.

Data from the included publications were collected independently by two researchers (TDC, JB) in pre-defined excel files and compared afterwards until consensus was achieved. In case of disagreement, the opinion of SG was requested. Variables collected included Country/region, year of study, population size/coverage, intervention type and specifics, Incidence/prevalence data, other relevant intervention outcomes, follow-up period, randomness, presence of a control, adverse events, limitations, references.

## 3 Results and discussion

**Fig. 2:**
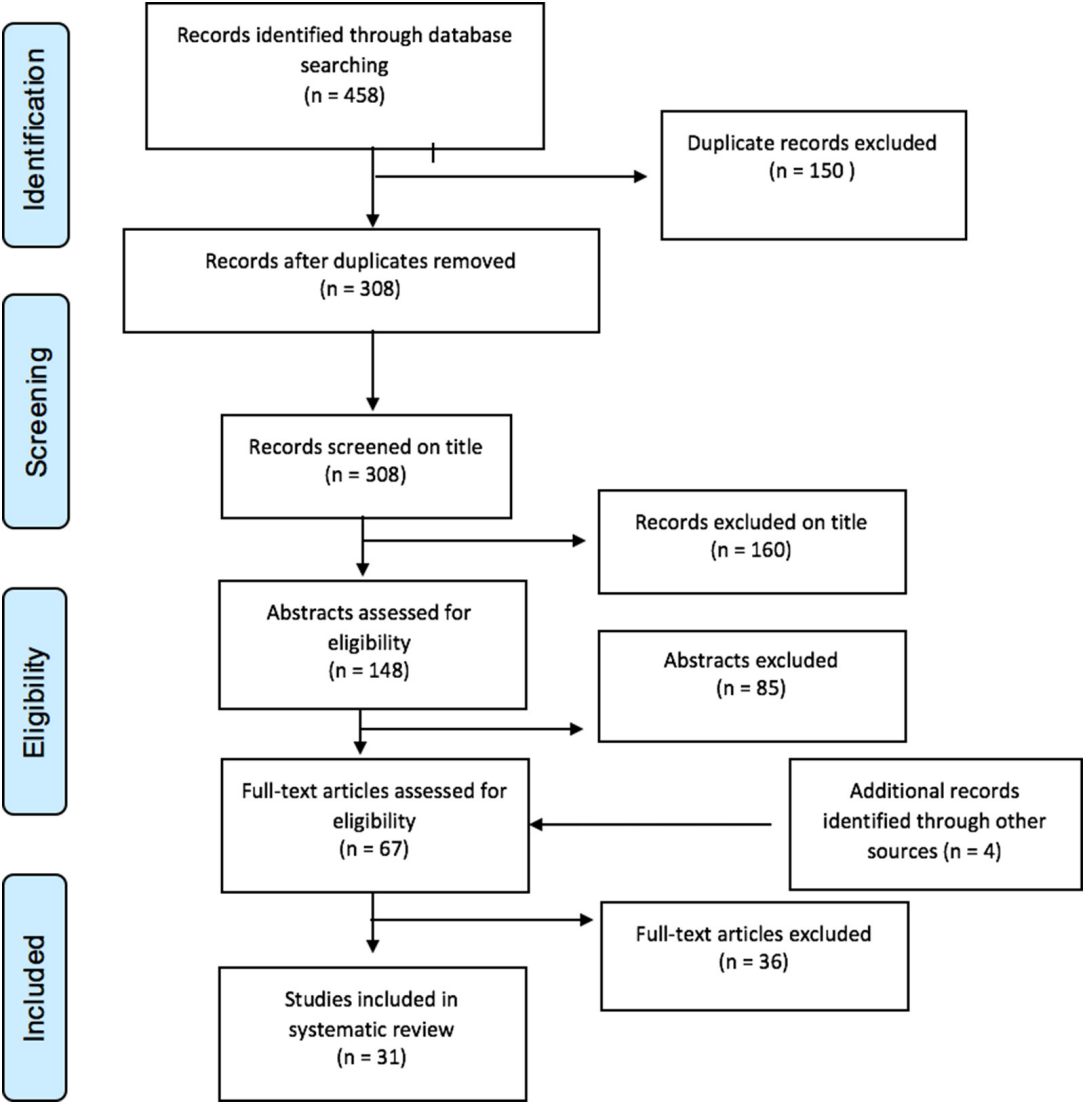
PRISMA flow chart of the selection process.

The database search entry resulted in the identification of 458 records of which 31 were included after removing the duplicates, screening the titles, abstracts and full texts, based on the above-mentioned exclusion criteria and including additional records. Of the 31 papers included in this analysis, 13 were field trials relating to the control of *T*. *solium*. Eighteen other articles contained experimental (non-field) data, mathematical models, data relating to aspects of NTD control that were directly relevant to the control of *T*. *solium*, meeting reports and national/international guidelines and strategies (Fig. 2). Field and experimental studies are discussed and included in tables classified by type of intervention.

A number of identified publications described mathematical models. Mathematical models can be run in a quick way, are cheap to implement and render rapid theoretical insights in which intervention tool, which algorithm or which stepwise approach of interventions will prove most useful in obtaining control or elimination. Five models, published since 2014 were identified. Three of them focus on single intervention options and combinations thereof targeting the human populations as well as the pig intermediate host (Braae et al., 2016a; Johansen et al., 2017; Winskill et al., 2017). The fourth modelled the outcome of three interventions targeting the pig population (Lightowlers and Donadeu, 2017). A last model assessed the risk of eating pork meat in western Kenya and the effect of meat inspection on the outcome (Thomas et al., 2017). These models are extensively described and discussed in Dixon et al. (submitted) and will therefore not be exhaustively repeated here. Depending on the model and inputs/parameters used, variations in output are observed between the different models interpreting the same interventions. Nevertheless, most models corroborate the finding that for a sustained, long term control, multiple interventions are needed. The results of this review will focus on the outcomes of field trials with reference to certain model outcomes when relevant.

### 3.1 Human treatment

When the WHO included *T*. *solium* cysticercosis as a NTD in 2010, MDA was recommended as the primary intervention strategy against TS (WHO, 2010). The systematic review of Thomas (2015) concluded that reduction of transmission over a short period is achievable through human Mass drug administration (MDA) (using niclosamide (NCZ) or praziquantel (PZQ)) as a single or a combined control option. Recent mathematic models suggest that a single round of human treatment is not sufficient to obtain sustained control (Braae et al., 2016a; Johansen et al., 2017; Winskill et al., 2017). Therefore, the scope of interest has moved towards finding a combination and algorithm of multiple strategies and all recent field studies on single human MDA (Table 1) included multiple treatment rounds. Two recent field trials (Braae et al., 2016b, Braae et al., 2017; Ash et al., 2015) studied the potential of integrating *T*. *solium* control into larger NTD MDA programs. These strategies fit in a larger One Health approach as they have the potential to tackle more diseases at once and might improve cost-benefit ratios. Braae et al., 2016b, Braae et al., 2017; (Table 1) assessed the effect of the national schistosomiasis control program (MDA of PZQ (40 g/kg) in school aged children (SAC)) in combination with track and treat of TS cases, on the prevalence of TS and PCC. A significant decrease of TS and PCC was found after three rounds, whereas a two-round MDA did not show a significant result. TS significantly dropped in both children and adults in the three round MDA system, based on the copro-Antigen ELISA, indicating that a single approach intervention of multiple rounds of MDA targeting a proportion of the population (SAC) can impact on transmission and spill-over into the pig population and the untreated adult human population. Though the latter could not be consistently proven (Braae et al., 2017) on short term. Also, the contribution of additional track and treat of TS cases in the whole population to the decrease in prevalence cannot be calculated, questioning the efficacy of multiple rounds of MDA treatment to SAC without the additional track and treat. Co-endemicity of schistosomiasis and *T*. *solium* (Braae et al., 2015a) and increased availability of donated PZQ for the treatment of schistosomiasis support an integrated approach for both parasites, nevertheless, the higher dose recommended for the treatment of schistosomiasis might increase the risk of epileptic seizures in NCC persons. Therefore, co-endemicity maps should be produced at district/village level to assess the risk and benefit of tackling both diseases at once. Also, the impact of a lower number MDA rounds in low *Schistosoma* prevalence areas needs to be assessed, on the short and long term.

**Table 1:** Summary on the recent field trials involving human treatment. (*ALB*: *albendazole*, *BL*: *baseline*, *CI*: *confidence interval*, *cov*.: *coverage*, *d*.: *day*(*s*), *HH*: *household*, *MDA*: *mass drug administration*, *m*.: *month*(*s*), *NCZ*: *niclosamide*, *PCC*: *porcine cysticercosis*, *pi*.: *pigs*, *pp*.: *people*, *PZQ*: *praziquantel*, *SAC*: *school aged children*, *STH*: *soil transmitted helminths*, *TS*: *taeniasis*, *X*: *no results of this variable*, *y*.: *years*, ≠: *difference*, ♂: *male*). *The two articles indicated with* * *were written about the same field study*.

Country, region	Year	Population size/coverage	Intervention	Incidence/Prevalence		Follow-up period	Random	Control study arm	Citation
				TS	PCC				
Peru, Surpampa and Santa Ana	?	1058 pp. (Surpampa), 753 pp. (Santa Ana).	4-monthly ring-screening and tx. (NCZ) TS cases, 100 m. from heavily-infected PCC pi.	Adjusted prev. TS at 16 m.: ±4 × lower in the intervention arm compared to control at 16 m.(prev. 0.28, 95% CI 0.08–0.91).	-Intervention- arm: 41% reduction (12 m.) compared to BL (incidence rate ratio 0.59, 95% CI 0.41–0.87) -Control: unchanged.	4, 8, 12, 16 m.	Selected on similar size/terrain/visible presence free-range pi.	Yes	(O'Neal et al., 2014)^1^
Tanzania, Mbozi and Mbeya district.	2012–2015	14 villages, 1500 pp., 400 pi. per district, per survey.	- Annual MDA rounds to SAC (2 Mbeya, 3 Mbozi): PZQ (40 mg/kg). - Track and treat of TS cases (NCZ).	- Mbozi: prev. adults 4,1% to 1,8% (*p* = 0.031); prev. children: 2.3% to 0.1%. - Mbeya: no significant result.	- Mbozi: 13% to 8% (*p* = 0.002) Mbeya: no significant result.	BL, 3 (pi. only), 6 12, 24 and 32 m.	X	No	(Braae et al., 2016b)^2^*
Tanzania, Mbozi and Mbeya district.	2012–2014	14 villages, 305,319 pp., (Mbeya), 446,339 pp. (Mbozi) and 31,190 pi. (Mbeya) and 117,483 pi. (Mbozi). MDA cov.: 34% of total population. Track and treat cov.: 9% of TS cases.	SAC MDA, 1 (Mbeya) or 2 (Mbozi, annual) round (PZQ, 40 mg/kg), track and treat TS cases (NCZ, total sampled population).	R0: ≠ between Mbozi (3%) and Mbeya (1.5%) (*p* = 0.007) R1: - Mbozi: 3% to 2% (*p* = 0.024). - Mbeya: 1,5 to 0.3%, (*p* = 0.004), ≠ between two districts (*p* < 0.001). R2 - Mbozi: 2% to 0.8% (*p* < 0.001) - Mbeya: 0.3%, to 0.5% (*p* = 0.051), no ≠ between two districts (*p* = 0.51).	X	BL, 12 m. and 22 m. after first MDA.	Selected by knowledge/PCC presence.	No	(Braae et al., 2017)^2^*
Southeast Asia, Lao PDR	Nov. ‘13- Apr. ‘14	298 and 295 pp., 60 HHs, 64% cov.	2 MDA rounds (ALB 400 mg, 3 d., interval of 5 m.).	- TS decrease by 79.4% (MDA1). - prev. steady during inter-treatment interval - 100% decrease (MDA2).	**STH**	BL, 1 m. and 5 m. after MDA 1. 1 m. after MDA 2.	No	No	(Ash et al., 2015)^2^

In the study of Ash et al. (2015) ( Table 1), the effect of two MDA rounds of a triple albendazole treatment, undertaken five months apart, on the prevalence of TS and soil transmitted helminths (STHs) was measured. Albendazole was the drug of choice because of its broad spectrum efficacy against both *T*. *solium* and STHs (Horton, 2000; Steinmann et al., 2011), absence of severe adverse effects (as opposed to PZQ) and acceptability to the government. A significant reduction in levels of both TS and STH prevalence was achieved, but the increased STH prevalence, detected between MDAs, reflects the problem of reinfection through inadequate behaviour (open defecation) and lack of sanitary conditions. Additional measures which support behavioural changes (such as health education), improved sanitation, and sustained chemotherapy programs, which may also need to tackle reservoir hosts, may therefore be needed to prevent transmission and obtain sustained results.

MDA entails treatment of a majority of non-infected people, implying spilling of resources and associated risks. Selective treatment of TS cases is considered an alternative, however remains difficult, bearing in mind the diagnostic limitations identifying tapeworm carriers due to vague/no clinical signs or symptoms and the unavailability of sensitive, specific, field-friendly detection methods in endemic areas, as well as the cost linked to this procedure (Alexander et al., 2011). The epidemiological basis of removing carriers out of the population is however undeniable and a recent mathematical model by Winskill et al. (2017) calculated an annual track and treat strategy to be very effective. Research on development of better diagnostic tools is currently ongoing and if successful, the feasibility of this strategy might improve in the upcoming years. Focus based treatment founded on medical or veterinary data was proposed earlier as another cost-effective strategy (Pawlowski, 2008), however is also not feasible due to inadequate record-keeping and disease surveillance. Alternatively, focus based ring screening and treatment of human (and pigs) within a 100 m radius of heavily infected pigs (identified by tongue palpation), was recently evaluated (O'Neal et al., 2014; Table 1). Studies by Pray et al., 2016, Pray et al., 2017 and Thomas et al. (2013) on pig roaming practices and exposure to human defecation support interventions targeting the areas immediately surrounding heavily-infected pigs where *T*. *solium* transmission is most likely to occur. The ring-screening and treatment reduced the transmission of *T*. *solium*. A 41% reduction in sero-incidence among pigs born during the intervention period was observed, as well as a nearly four times lower TS prevalence in the intervention community, though the latter outcome was not significantly different from the control area (O'Neal et al., 2014). In addition, considerable variation in sero-incidence in the intervention village was measured, the study was not randomized, only two villages were included and PCC infected pigs might have been overlooked, as tongue palpation is often only positive when the pigs are heavily infected. These limitations suggest that while it seems the approach may be effective and practical for regions where resources are limited, testing this strategy in larger studies with longer follow-up time is necessary to validate the approach and determine the long-term effects.

### 3.2 Health education

The Landscape Analysis (Thomas, 2015) stated that health education (HE), as many other measures, as a stand-alone strategy is not sufficient in terms of efficacy and cost-benefit to control *T*. *solium*. *S*till, studies have determined that by changing attitudes and practices via HE based on sound theoretical frameworks (WHO-EMRO, 2016), the prevalence of *T*. *solium* cysticercosis/taeniosis can be reduced (Sarti and Rajshekhar, 2003; Ngowi et al., 2008). Since 2013, six articles related to HE were published. Four on field trials (Table 2.) and two on non-field trials.

**Table 2:** Summary on the recent field trials involving health education. (av: average, BL: baseline, Child.: children, ctl.: control, HCC: human cysticercosis, int.: intervention m.: month(s), PCC: porcine cysticercosis, pi.:pig(s), TS: taeniasis, X: no results for this variable, y.: years, ≠: difference).

Country, region	Year	Population size/coverage	Intervention	Improvement				Follow-up period	Random	Control	Citation
				Knowledge	Practice/attitude		Active HCC				
					+	–					
Tanzania, Mbulu	2010–2012	2700 child., 60 schools	Video show, education by trained teacher, leaflet.	Increased 10% (int.) and 6% (control) after 6 m. on TS, PCC, HCC, epilepsy.	Condemning infected meat (int.), how best to raise pi. (control).	Contacting a veterinarian for infected pi.	X	Immediately, 6 m., 12 m.	Yes	Yes	(Mwidunda et al., 2015)
Tanzania, Mbeya	2014	79 professionals	Presentation, work on computer with ‘The vicious worm’.	Improved, immediately (*p* = 0.001) and 2w. after (p < 0.001).	efficient, simple, appealing.	computer-based design, suggestion: supplement leaflets	X	Immediately, 2w.	No	No, BL used as control.	(Ertel et al., 2017)
Zambia, Katete District	Jul’ and Nov’ 16	3 primary schools, 99 students	1/2-day workshops using ‘The vicious worm’.	High at BL (av. 62%), significantly improved immediately after (*P* < 0.05 (part of the questions in 1 school), (*P* < 0.001 for all questions in 2 schools).	X		X	Planned in study neighbourhood	No	No, BL used as control.	(Hobbs et al., 2018)
Burkina Faso, Boulkiemdé, Sanguié and Nayala	2011–14	60 villages (2 excluded), 4645 eligible pp.,	screening and discussion of a movie, and a Self-esteem, Associative strengths, Resourcefulness, Action planning, Responsibility (SARAR) approach via the Participatory Hygiene and Sanitation Transformation (PHAST) model.	X	Increase of proportion HHs with latrine.	No difference in pi. penning	Effective in 2/3 provinces. Decrease in cumulative incidence: ratio = 0·65, (95% C.I. 0·39–1·05) and decrease in prev. proportion ratio = 0·84 (95% C.I. 0·59–1·18). from BL to after int.	BL, 18 m. (before intervention), 36 m (18 m. after int.).	Cluster-randomization	Yes	(Carabin et al., 2018)

In 2014, ‘The Vicious Worm’ was introduced as a computer-based *T*. *solium* education tool (Johansen et al., 2014), to be included as an evidence-based specific control measure in any control program. ‘The Vicious Worm’ aims to bring a simple and meaningful message, tackling various difficulties (e.g. reaching stakeholders across disciplines and sectors, clarifying the complicated lifecycle) and misunderstandings (e.g. the association of the disease with superstation and witchcraft or the belief that you can't get the disease if you don't eat pork). From its introduction on, the computer-based education program has been assessed for its efficacy in knowledge uptake, cultural acceptability and compliance in different test groups and information of these assessments has been used to adjust and update the tool. A first assessment of ‘The Vicious Worm’ education tool on knowledge uptake and attitude towards the program among professionals from the health and agricultural sectors in the endemic Mbeya area in Tanzania was published by Ertel et al. (2017), based on questionnaire surveys. The study subject's overall knowledge was significantly improved both immediately after (77% (95%CI:67.7–86.3), (*p* < 0.001)) and two weeks after (70%(95%CI:59.7–80.3), (p < 0.001)). The knowledge regarding acquisition and transmission of *T*. *solium* infections and the relation between PCC, HCC and TS was not significantly improved. Positive attitudes towards the program were found after focus group discussions, only the computer-based design was mentioned as a limitation for some study subjects and leaflets as supplement for rural areas were suggested. In contrast with the studies by Maridadi et al. (2011) and Mwidunda et al. (2015), neither gender nor educational level, were shown to be significant factors for knowledge uptake.

Hobbs et al. (2018) made an assessment of the knowledge uptake among primary school students, also based on questionnaires, and found an improved knowledge score immediately after the workshop. While key messages to prevent transmission were better understood, details on the parasites lifecycle were less well captured as observed with the professionals (Ertel et al., 2017). Follow-up evaluations one year after the administration of ‘The Vicious Worm’ have been conducted and are currently being analysed (S. Gabriël, personal communication).

These findings reflect the complexity of the parasite's life cycle and the need to simplify concepts. Nevertheless, ‘The Vicious Worm’ was found to be an effective tool to improve knowledge, regardless of gender, level of education or experience with *T*. *solium* and computers. The tool has now been translated into Swahili (Trevisan et al., 2017), while other languages are planned. Other advantages include the reduction of teacher training costs, provision of uniform education and possibility to continuously improve the computer-based program. Of course, studies on behavioural changes and the effect of ‘The Vicious worm’ on the reduction in disease burden should be conducted in the future.

An optimal HE strategy for the endemic region of Burkina Faso was singled out using the PRECEDE-PROCEED (Predisposing, Reinforcing, and Enabling Constructs in Educational Diagnosis and Evaluation) model by Ngowi et al. (2017). The model is a planning model based on the idea that an educational diagnosis precedes an intervention plan and enhances the potential appropriateness and effectiveness of interventions. The main problems identified in Burkina Faso area were the lack of knowledge on TS and cysticercosis, and problems maintaining hygiene and sanitation in combination with the practice of free-roaming animals. The practice of free-roaming pig husbandry was perceived as an economically rational strategy for an impoverished population, which is unlikely to change. Based on the PRECEDE model, a multicomponent educational intervention approach to tackle the identified issues was developed. This intervention included a SARAR (Associative strengths, Resourcefulness, Action planning, and Responsibility) and PHAST (Participatory Hygiene and Sanitation Transformation) approach model (WHO, 1997), including community based training sessions on building latrines, latrine use and open defecation, supplemented by a 52 min. film, a discussion summarizing and identifying the key messages of the film and a comic booklet to improve knowledge about *T*. *solium* transmission and the benefits of its' control. Results of this first cluster-randomized, community-based, drug free study in sub Saharan Africa on the cumulative incidence and prevalence of active HCC, showed a decrease in both outcome measures and the efficacy of the intervention in two of the three provinces included in the study (Carabin et al., 2018; Table 2). The differences in ethnic groups and social structures might have had a role in the non-efficacy found in one province. Overall, this study shows that community-engaged participatory interventions have potential as a low-cost control intervention strategy for cysticercosis in some low-resource settings, with possibilities for implementation at a larger scale. However, area depending adaptations might be necessary to ensure the effectiveness.

When implementing a HE strategy, it is also important to consider the potential of the target population in terms of knowledge uptake and change of attitudes and transfer of these to the rest of the community. HE was found to be useful in controlling TS and cysticercosis as mentioned above, but trials so far, have mostly been targeting adults. Alexander et al. (2011) reported improved knowledge and practices after a HE session to school children in India, but the lack of a control group made the study unable to estimate the actual effect of the intervention. Elsewhere, school children have been found to be good targets for messages to control other health problems and also their quality as good knowledge carriers has been highlighted. Mwidunda et al. (2015) could demonstrate a 10% knowledge improvement on *T*. *solium* in school children after six months as well as a positive effect regarding practices towards the disease. A simultaneous improvement of 6% in the control group was attributed to the administration of the questionnaire twice in a short interval, which might have stimulated interest in the topic and led the children to think about their answers more carefully. Also, differences between primary and secondary school pupils were found, suggesting a higher exchange of information among secondary school pupils, as they interact among themselves and across schools in contrast to children of primary schools, who use most of their free time playing games. Additionally, older school pupils are more respected, which may give them more time to meet with their peers for socialization. The study also found that increased age was associated with positive attitude to condemn infected pork and consult with a veterinarian for infected pigs as well as better scores for the knowledge part.

Future studies should assess the length of time to which the acquired knowledge would persist as well as its contribution to behaviour change and reduction in disease burden.

### 3.3 Pig oriented interventions

Avoiding pigs consuming human stool can fairly straightforward be tackled by confining them. However, socio-anthropological research including focus group discussions conducted in eastern Zambia by Thys et al. (2016) identified a number of barriers to pig confinement. The study revealed that pig confinement is currently not perceived as an acceptable method to control PCC by farmers in Eastern Zambia, based on the pigs' role in society (financial, agricultural and traditional), environmental aspects (feed supply, presence of bush, wood use priorities, rainy season) and the distribution of the management tasks among the family members owning pigs (feeding, building kraal, seeking care). Indeed, gender plays an important role because women, and also children, seem to have a higher perception of the risks but lack power in terms of economic decision-making compared to men. Addressing men concerning this matter during HE sessions might be a good strategy. Nevertheless, even if negative aspects/health risks of free-range pig keeping are perceived, people seemed ready to take the risk for socio-economic reasons.

Also, in a sero-prevalence study Braae et al. (2014) remarked that confined pigs did not have a lower PCC sero-prevalence compared to free-roaming pigs. Elevated pens, pens with a dirty floor, open latrines and feeding potato peels were identified as PCC infection risk factors in a follow-up case-control study (Braae et al., 2015b). Improving pig management should therefore not be limited to confinement, but should also address these risk factors.

Besides confinement, two measures have been evaluated to break the parasite life cycle on the pig's level, being treatment and vaccination.

#### 3.3.1 Pig vaccination

To this date, SP3Vac and TSOL18 have progressed the furthest and have been tested under experimental and field conditions (Aluja et al., 1999). The TSOL18 (Cysvax®) vaccine was reported to be registered and available for sale upon November 2016. The influx of new born susceptible piglets into the vaccinated population remains a challenge when envisaging vaccination campaigns, as pigs in rural areas do not have a breeding season, and vaccination of young piglets is thought ineffective due to presence of maternal antibodies. As a solution, treatment of pigs (oxfendazole, OXF) to eliminate active infection combined with vaccination (TSOL18) for longer term protection against re-infection, was proposed and proven efficient by Assana et al. (2010). The use of this strategy has previously been recommended as a short-term vertical control program followed by a long-term sustainable horizontal program with the potential for eradication in two seasons (Assana et al., 2013). Though effective (99% efficacy in protecting pigs against infection when used in conjunction with a single dose of OXF (Gauci et al., 2013), both vaccination and chemotherapy in pigs should be applied in a way that would be effective, feasible and sustainable under field conditions, taking local pig management practices into account. Lightowlers (2013) therefore identified a schedule involving four-monthly treatment of pigs with both TSOL18 and OXF aiming to achieve a high level of disease control, while minimizing the number of interventions that would be required on annual basis. To test whether this long interval between the first and second immunization, as applied in the suggested schedule, results in sufficient protection, an assessment of the specific antibody responses in pigs immunized with the TSOL18 vaccine was made by Lightowlers et al. (2016) by altering intervals between the two immunizations (4,8,12,16 or 20 weeks) (Table 3A.). Results of this trial show antibody responses up to intervals of 20 weeks and suggest that immunizations with TSOL18 given at approximately three-monthly intervals should provide continuing protection after the second injection. While antibody titres have been previously used to evaluate responses to the vaccine that are associated with protection (Kyngdon et al., 2006; Assana et al., 2010), a true efficacy claim of a three- or four-monthly based TSOL18 vaccination/OXF treatment schedule requires field trials assessing at least PCC levels to prove a reduction of transmission on the pig level. A four-monthly vaccination (TSOL18) and OXF treatment scheme, integrated in a short-term elimination strategy together with HE and Human MDA was recently tested in a large-scale field study in Zambia (personal communication, S. Gabriël).

**Table 3:** A. Summary of one field trial on pig vaccination. (Ab: antibody, im: intramuscular, m.: months, pi.: pigs, w.: weeks, wo.: weeks old, X: no information of this variable, ♂: male, ♀: female) B. Summary of one field trial on pig treatment. (nr.: number, OXF: oxfendazole, PCC: porcine cysticercosis, pi.: pigs, po: per os, TCBZ: triclabendazole, tx.: treatment, w.: weeks, <: smaller than).

A.								
Vaccine	Type	Population	Protocol	Protection (anti-TSOL18-specific IgG titers)	Follow-up period	Random	Control	Citation
TSOL18	Experimental	50 Landrace-Pietran pi. (12 wo., half ♂, half ♀.)	2 doses, im. (neck), 4,8,12,16 or 20 w. apart.	- 100% response - No diminution in Ab responses for doses up to 20 w. apart. - Titers in groups receiving the 2nd immunization >4 m. apart developed higher mean ab titers than pigs receiving their 2 doses 4 w apart. - Animals immunized at an interval of 12 w. had 3/1 times the Ab titer of those immunized at an interval of 4 w.	X	Yes	Yes	(Lightowlers et al., 2016)


B.									
Anthelmintic	Type	Population	Cyst burden (limb muscles, degenerated, calcified and viable)	Cyst appearance	Adverse effects	Follow-up period	Random	Control	Citation
TCBZ/OXF	Experimental tx. of naturally infected PCC pigs.	18 pi. (Huancayo, Peruvian highlands), 6 tx. TCBZ 1 × 30 mg/kg po, 6 1 × 30 mg/kg OXF po, 6 1 x sugar water po (control group).	- Control: 1658 (131 - 2575) cysts. - TCBZ: 1414 (511 - 4052) cysts - OFZ: 259 (38 - 1953) cysts. - nr. of cysts: OXF group<TCBZ group (P < 0.001).	- TCBZ: mild - moderate inflammatory response around cysts - OXF: 100% degenerated cysts, severe inflammatory reaction.	None	17 w. post-tx.	Yes	Yes	(Vargas-Calla et al., 2016)

#### 3.3.2 Pig treatment

Several studies on the efficacy of anthelmintic treatments against PCC have been performed in the past (i.e. albendazole, flubendazole, fenbendazole, nitazoxanide, and PZQ) (Mkupasi et al., 2013). Almost all tested anthelmintic drugs are active against adult stages of cestodes, nematodes and trematodes, but the challenge has been to identify drugs effective against the larval stage (Gonzalez et al., 2012). Among efficacious larval stage drugs are OXF, albendazole and PZQ (Gonzalez et al., 2012; Flisser et al., 1990). OXF (30 mg/kg) remains the drug of choice, due to the requirement of multiple doses for albendazole and praziquantel, which is impractical in the field (Mkupasi et al., 2013) and the reports of adverse effects as anorexia, lethargy, prostration and death (Gonzalez et al., 2012). A shortcoming to OXF is lack of 100% efficacy against cerebral cysts (Gonzalez et al., 1998; Sikasunge et al., 2008), although it is thought that consumption of undercooked pork brain is not common in most endemic areas (Gonzalez et al., 1998). OXF 10% (Paranthic®) was registered in Morocco in 2013 and is currently the only registered drug for PCC treatment. Registration processes for both OXF and the TSOL18 vaccine are currently underway in other endemic countries.

The widely used triclabendazole (TCBZ), another member of the benzimidazole family, next to OXF and albendazole, is efficacious against adults and larval parasite stages such as *Fasciola* spp., *Paragonimus* spp., some nematodes and cestodes (Coles, 1986; Keiser et al., 2005; Richter et al., 2013). On these bases, Vargas-Calla et al. (2016) recently evaluated the efficacy of TCBZ against PCC (Table 3B). TCBZ in a single dose of 30 mg/kg was not efficacious against *T*. *solium* PCC in the study as there were no apparent differences in cyst burden or appearance between the TCBZ treated group of animals and the control group on necropsy. Subsequent microscopic evaluation however, showed mild inflammatory reactions, which might indicate efficacy of a higher dosage or multiple dose administration. Multiple doses of TCBZ were previously reported to be efficacious against *Echinococcus multilocularis* metacestode cystic larval stages in an in vitro study by Richter et al. (2013), but would be impractical under field conditions.

A challenge inherently linked to the use of an anthelmintic is the withdrawal time after treatment before safe consumption of the meat.

### 3.4 Sanitation

Improvement of basic sanitation, hygiene and HE has proven to be an effective strategy for parasitic and infectious diseases transmitted by faeces (Fleury et al., 2013). Community Led Total Sanitation (CLTS) is an innovative community-based sanitation program which aims to reduce open-air defecation through the construction of latrine pits. It is assumed that CTLS will lead to the control of poor sanitation-related diseases, including HCC/PCC. In 2007, UNICEF piloted in conjunction with the Government of Zambia the CTLS approach in the southern province of Zambia, with a promising outcome as sanitation coverage increased from 23 to 88%, and 75% of the villages were open defecation free. Afterwards, ‘The 3 million People Sanitation Program’ was launched by the Minister of Local Government and Housing in Zambia. Twelve districts were included in a pilot study during including Katete in the Eastern Province. A preliminary evaluation of the effectiveness of CLTS in the Eastern Province of Zambia by Bulaya et al. (2015) (Table 4) could however not repeat these promising results. Eight months after the implementation of CTLS in nine villages, sero-prevalences of PCC did not significantly improve and the knowledge, attitudes and practices did not substantially change. Not all households constructed latrines, and the introduction of latrines did not guarantee the use of them. The study also identified a number of cultural practices and traditional beliefs hampering the latrine usage corroborating findings of Thys et al. (2015) who conducted a socio-anthropological study based on focus group discussions in a neighbouring district. Occupants of a household would not use the same latrine in relationships such as adults with children or in laws with parents. Latrines were not constructed in every household because of the convenient use of existing latrines in the neighbourhood. Moreover, Thys et al. (2015) found that mostly men are responsible for building latrines and mostly men prefer open defecation. Sanitation programs should therefore be combined with health education, not only addressing the health benefits, but also focussing on the local context and the sanitary-related taboos, targeting also the men, to achieve an increased usage of the latrines.

**Table 4:** Summary on the outcome of the Total Led Control Sanitation (CTLS) program by Bulaya et al. (2015). (AFS: African swine fever, BL: baseline int.: intervention, m.: month(s), PCC: porcine cysticercosis, pi.: pig(s), pos.: positive).

Country/region	Year	Population	Intervention	Improvement		Knowledge	Follow-up	Random	Control
				PCC (serum Ag ELISA)	Sanitation practices and attitudes				
Zambia, Katete District.	Apr.- Jun. ‘12.	65, 865 pi. 48,417 pp. 104 pi. (pre-int.), 275 pi. (post-int.). 64 (pre-int., 19% response rate) and 89 respondents (post-int. 26% response rate)	CLTS: construction of pit latrines.	BL: 14 pos. (13.5%) Post- int.: 45 (16.4%) pos. (p: 0.473).	- Crop season: pi. are kept more in pens. - More toilets: 43 (67.2%), pre- and 74 (83.1%) post. Intervention. - Increase of latrines presence: pre (43, 67.2%) - and post-int. (74, 83.1%) (*p* = 0.027). -Latrine usage: no increase: 41 (93.2%) at BL and 62(84.9%) post-int. giving a net increase of only 21 latrines (*p* = 0.15). This means that there has only been a 33.9% increase in toilet usage (p = 0.15). - Home slaughter: common practice - No change in selling pork with cysts pre- and post-int. (*p* = 0.679) - AFS: most important pig disease (p = 0.00)	- 80% had heard about/observed PCC.	8 m.	No, villages were chosen on certain criteria.	Yes

### 3.5 Combinations

Mathematical models (Kyvsgaard et al., 2007; Braae et al., 2016a; Johansen et al., 2017; Winskill et al., 2017) suggest that both pig and human treatment are required to obtain a rapid and sustainable impact on disease transmission and presence. Up till now, three strategies have been attempted or modelled, including the combination of pig MDA/vaccination and human MDA (Garcia et al., 2006; Garcia et al., 2016; Braae et al., 2016a; Okello et al., 2017; Johansen et al., 2017; Winskill et al., 2017), the combination of pig vaccination and human health education (De Aluja et al., 2012) and the combination of pig vaccination and human MDA (only modelled by Kyvsgaard et al. (2007)). In this review, two field trials on the combination of human and pig MDA combined with pig vaccination are included, one measuring the effect on TS prevalence and the other on elimination of transmission (Table 5).

**Table 5:** Field trials on combination strategies. (C.I.: confidence interval, cov.: coverage, d.: day(s), HH: household, m.: month(s), MDA: mass drug administration, NCZ: niclosamide OXF: oxfendazole, pi.: pig(s), pp.: people, po: per os, prev.: prevalence, tx.: treatment, vacc.: vaccination, y.: year).

Country, region	Year	Population size and coverage	Intervention	Outcome	Follow-up period	Random	Controlled	Citation
Peru, Tumbes	2007?-?	107 villages, 81,170 pp. (84.7% cov.), 55,638 pi.	Human MDA (NCZ, 3 rounds) & pi. MDA and vacc.(OXF, every 2 m. + TSOL18, 2 rounds of 2 vacc.).	3/342 pi.: live, non-degenerated cysts, no infected pi. in 105/107 villages. 1 y. later: 7/310 pi.: live non-degenerated cysts, no infected pi. in 11/17 villages.	1 m., 1 y.	No	No	(Garcia et al., 2016)
Northern Lao, Mai District	Oct. ‘13 – Jan. 15’	300 pp. (55 HHs), 63% (MDA 1) and 65% (MDA 2) cov. 414 pi. (90% cov.).	2 rounds of 3d. human MDA (albendazole 400 mg, 10/13 and 03/14.), 3 rounds of TSOL18 vacc. & po pi. tx. (OXF, 30 mg/kg, 10/13, 03/14 and 10/14), repetition after 1 m.	78.7% decrease in TS population prev. From 30.6% (95% C.I. 25.5–38.9%) to 6.5% (95% C.I. 3.4–9.5%). Significant reduction.	12 m.	No	No	(Okello et al., 2017)

Okello et al. (2017) observed that a combination of both human and porcine MDA can result in a significant decrease in human TS levels in a relatively short period of time. The study was the first to test this in a Southeast Asian context and provided the first data on the impact upon the adult parasite in the human host. Two rounds of community MDA with three consecutive doses of albendazole 400 mg at 6 months interval, combined with pig vaccination (TSOL18) and treatment (OXF) followed by a repeat pig treatment one month later for three iterations (first two combined with the human MDA). A significant (*p* < 0.0001) TS reduction was measured, prevalence, determined via copro-Ag ELISA, dropped from 30.6% (95% CI: 25.5–38.9%) before the first intervention to 6.5% (95% CI: 3.4–9.5%) after the last intervention. Lack of data on PCC and sufficient coverage were limitations of the study, though the human coverage of 63–65% of the population was compensated by a coverage of 90% of the pig population. However, transmission by pig meat can even occur after implementation of this strategy through consumption of pigs before slaughter age (<6 m) or pigs from outside the intervention zone. This was confirmed by three new cases of TS, who had at least received one full course of albendazole treatment.

A similar approach was used by Garcia et al. (2016), with the aim of eliminating *T*. *solium* in an endemic area of Northern Peru. This three-phase program, started off testing and comparing elimination strategies in terms of feasibility and effectivity of interrupting the transmission of *T*. *solium* infection in 42 villages. In a second phase the two most promising strategies from intervention one, mass treatment, and mass screening and treatment (both with or without vaccination of pigs) were compared in 17 villages. In a third and final phase, the final strategy of human MDA combined with pig MDA and vaccination was implemented in the entire rural region of Tumbes (107 villages). The study achieved interruption of transmission in 105 of 107 villages through a one-year attack approach and elimination persisted in most areas for at least one year without further intervention. The one-year study-design was however not controlled and very intensive, including short intervals between rounds of MDA and vaccination. These aspects raise the question whether the elimination goal is a practical and economically viable target, especially for resource poor countries in sub Saharan Africa.

This review has focussed on the English literature, which inherently can lead to missed information available in other languages only.

## 4 Conclusion

Results from recent field studies confirm the need to implement combinations of interventions or multiple rounds to obtain rapid and substantial reductions in transmission, though most studies have important limitations in terms of inclusion of control arms, coverage, sample size, variations in outcomes, lack of randomization etc. The quick impact of the combination of human and pig treatment as predicted by the models was confirmed in a South Asian context by the study of Okello et al. (2017) and in Peru by Garcia et al. (2016). Moreover, elimination of transmission through a one-year intensive program, combining human and pig treatment/vaccination was determined achieved by Garcia et al. (2016).

Pig vaccination, combined with treatment was often mentioned as a very suitable intervention. Recent experiences in remote areas in Zambia, indicate that this might be strongly area and pig management dependent. The fast turnover in pig populations, reluctance of farmers to inject their animals (Hobbs et al., 2018 in preparation), practical constraints in cold chain storage and transport, combined with poor veterinary services renders the use of the vaccine difficult in certain poor remote areas. Integration of *T*. *solium* vaccination in another disease control program, that is perceived as more economically relevant by farmers (e.g. if a vaccine would be available for African swine fever), might increase compliance of the farmers towards vaccination and render this a more plausible option.

Transmission is associated with habits that support transmission of the parasite. For example, lack of use of sanitary facilities, free-range pig husbandry, a lack of sufficiently sensitive meat inspection and a lack of encouragement around safe consumption are factors that might take more than one generation to alter. With this in mind, investing in long-term strategies focusing on broad-based knowledge, habits and hygiene that have advantages far beyond that of *T*. *solium* and providing structures and knowledge that are passed on to next generations will also be key in keeping transmission low, after control or elimination of the parasite is reached. The provisions of adequate sanitation and programs addressing sanitation related taboos are one of these measures (Bulaya et al., 2015; Thys et al., 2015). Recent studies also provide more data on the positive impact of specific health education and developed tools (Ertel et al., 2017; Ngowi et al., 2017; Carabin et al., 2018; Hobbs et al., 2018) providing opportunities for area specific community-engaged participatory interventions.

Field studies indicate that indeed control is achievable, though sustained, long term reduction will require a close monitoring including new interventions when the need arises. Migration of both people and pigs is an important factor to consider as both can be carrier of the parasite and (re)-introduce/increase the infection in controlled areas. Susceptible non-treated or non-vaccinated pigs carrying cysticerci might also be imported from nearby villages and consumed, monitoring of these Trojan pigs will be needed for sustained disease control. Collaboration with veterinary and medical services for active surveillance and meat inspection in endemic areas is therefore necessary for the sustained effect of any intervention. Furthermore, the contribution of the environmental component on the transmission of *T*. *solium* (e.g. survival of eggs) remains to be determined and considered.

Lastly, implementation of any intervention measure depends on the willingness of policy makers. Therefore, it is important to provide actual data on the prevalence and burden of *T*. *solium* and communicate this data at governmental level. Moreover, it is necessary to provide data on effective measures on the control of *T*. *solium* that are within reach, meaning financially and practically sustainable for a particular country or region. The proposed approaches should be tailored to the specific setting of the country, taking into account the specific transmission dynamics, cultural factors, financial means etc., to deal with the area specific challenges. The intensive strategies needed to obtain elimination raise the question whether this is a practical and economically viable target and which other measures are needed to sustain the effect.

## Declaration of interest

None.
